# Erythrocyte morphological symmetry analysis to detect sublethal trauma in shear flow

**DOI:** 10.1038/s41598-021-02936-2

**Published:** 2021-12-07

**Authors:** Antony P. McNamee, Michael J. Simmonds, Masataka Inoue, Jarod T. Horobin, Masaya Hakozaki, John F. Fraser, Nobuo Watanabe

**Affiliations:** 1 Biorheology Research Laboratory, Menzies Health Institute Queensland, Griffith University, Queensland, Australia; 2 Department of Life Sciences, Systems Engineering and Science, Graduate School of Engineering and Science, Shibaura Institute of Technology, Saitama, Japan; 3 Perth Blood Institute, West Perth, WA Australia; 4 Critical Care Research Group, University of Queensland and The Prince Charles Hospital, Brisbane, Australia; 5 Biofluid Science and Engineering Laboratory (Watanabe Lab), Department of Bio-Science and Engineering, College of Systems Engineering and Science, Shibaura Institute of Technology, Saitama, Japan

**Keywords:** Membrane biophysics, Optical imaging, Biomedical engineering, Blood flow

## Abstract

The viscoelastic properties of red blood cells (RBC) facilitate flexible shape change in response to extrinsic forces. Their viscoelasticity is intrinsically linked to physical properties of the cytosol, cytoskeleton, and membrane—all of which are highly sensitive to supraphysiological shear exposure. Given the need to minimise blood trauma within artificial organs, we observed RBC in supraphysiological shear through direct visualisation to gain understanding of processes leading to blood damage. Using a custom-built counter-rotating shear generator fit to a microscope, healthy red blood cells (RBC) were directly visualised during exposure to different levels of shear (10–60 Pa). To investigate RBC morphology in shear flow, we developed an image analysis method to quantify (a)symmetry of deforming ellipsoidal cells—following RBC identification and centroid detection, cell radius was determined for each angle around the circumference of the cell, and the resultant bimodal distribution (and thus RBC) was symmetrically compared. While traditional indices of RBC deformability (elongation index) remained unaltered in all shear conditions, following ~100 s of exposure to 60 Pa, the frequency of asymmetrical ellipses and RBC fragments/extracellular vesicles significantly increased. These findings indicate RBC structure is sensitive to shear history, where asymmetrical morphology may indicate sublethal blood damage in real-time shear flow.

## Introduction

Haemolytic blood damage, to varying degrees, is an unavoidable reality of all current generation mechanical devices indicated for cardiovascular circulatory support^1^. While haemolysis in artificial organs has several determinants, exposure to supraphysiological shear stresses greater than tenfold the upper limits of normal circulation is now considered the primary candidate to cause red blood cell (RBC) membrane rupture and destruction^2–6^. Recent investigations of shear-induced RBC damage have identified (through direct and indirect assessments) that the physical properties of the cytosol, cytoskeleton, and plasma membrane undergo functional and structural alteration in response to supraphysiological shear exposure^7–11^; this occurs prior to complete cell destruction/haemolysis^12^. Prolonged exposure to supraphysiological shear stress may even propagate abnormal RBC morphology^13^, and increase the presence of extracellular vesicles and membrane fragments^14–17^.

Due to infrastructure challenges, many of the reported shear-RBC observations have been performed following cessation of exposure to shear stimuli. The need remains for development of methods able to detect the onset of near real-time sublethal damage during shear flow. Watanabe et al.^18^ reported the use of a counter rotating shear system fit to a microscope for direct RBC observation. Using this apparatus, shear induced cell fragmentation and haemolysis was identified in real-time at 40-s of accumulated exposure to 288 Pa. Given the ability of this system to detect haemolytic ‘end-points’ in continuous shear flow, it is plausible that this apparatus could also have the sensitivity to investigate markers of blood cell deterioration prior to haemolysis.

Following exposure to supraphysiological shear stresses (below levels which induce detectable haemolysis), several investigations have reported that the morphology of RBC is substantially altered, with increased echinocytes^11,13,19,20^, dumbbell shaped RBC^21^, and large extracellular vesicles^18^ being observed. Extending recent work^18^ of the RBC shape change that occurs at the point of RBC lysis, and based on the physical changes that have been reported to occur to the RBC membrane^22,23^, we believe that alterations in cell morphology could provide a valid indicator of accumulated sublethal trauma in shear flow, where decreased cell stability would present with asymmetrical ellipsoidal morphology.

To detect RBC structural abnormalities in shear flow, an image analysis method would provide a powerful advancement for real-time analyses. Several researchers have developed and applied image processing methods to inspect RBC morphology at stasis in peripheral blood smears for the purpose of cell counting, static shape assessment^24^, analysis of RBC aggregates^25^ and pathological diagnosis of: malaria^26^, sickle cell disease^27^, and acanthocytosis^28^. Only few analysis methods have been developed for flowing RBC with ellipsoidal or parachute shapes^29,30^, with common methods of RBC deformability analysis utilising parameters based on various ratios employing the major and minor axes of the cell^31–35^. Currently, no image analysis technique has been proposed to detect morphologically aberrant RBC, indicative of sublethal damage, while still in shear flow.

Therefore, the aim of the present study was to: (i) investigate the onset of initial morphological alterations during exposure to supraphysiological shear stress that remains below the haemolytic threshold; and (ii) develop a new image analysis method for the detection of morphologically-aberrant RBC in shear flow.

## Materials and methods

### Blood sample preparation

Blood was carefully collected from two informed and consenting healthy male volunteers (aged 20–30 years) in accordance with the international guidelines for haemorheological laboratory techniques^36^. Briefly, venepuncture was performed using a 21-G needle and syringe, and subsequently transferred into a blood tube containing 1.8 mg∙mL^−1^ K_2_EDTA anticoagulant within 90 s of tourniquet application. To facilitate accurate shear stress quantification and control, the non-Newtonian properties of blood were overcome by 200 times dilution in a solution of polyvinylpyrrolidone (PVP; molecular weight 360 kDa) in phosphate buffered saline with a known viscosity of 30 mPa∙s, pH of 7.4, and osmolality of 290 mOsmol∙kg^−1^. All experimental protocols were completed within 4 h of initial blood collection. The experimental protocols of the present study were reviewed and approved by the Griffith University Human Research Ethics Committee (reference number: 2016/712) and the Shibaura Institute of Technology Ethics Committee (#17-003-1, #17-003-2), which conforms with the Declaration of Helsinki.

### Experimental procedure

The present study deployed a custom-built counter-rotating shear generator as previously described^17,35^. Briefly, this system contains a transparent Couette-type counter-rotating shear chamber mounted on an inverted microscope (IX73, Olympus Corp., Tokyo Japan) equipped with a high-speed CMOS camera (optiMOS™ sCMOS Camera, QImaging, Surrey, Canada), and illuminated by an halogen lamp (TH4-200, Olympus Corp., Tokyo Japan). Given the opposing shear profile of the counter rotating plates, RBC suspensions positioned towards the middle of the chamber should remain in a stable location. Small samples of RBC-PVP solutions may be loaded into this system, and directly visualised while being exposed to shear.

### Selection of shear stress conditions

To investigate whether the onset of RBC sublethal damage was associated with increased detection of aberrant morphology, the specific shear conditions chosen for the present study were carefully considered. Simmonds et al.^10^, identified RBC first experience shear-induced rigidification (i.e., subhaemolytic damage) following 300-s of exposure to shear magnitudes between 30 and 40 Pa. Thus, to examine shear-induced RBC morphology alterations, four discrete shear stresses were chosen centred around the ~35 Pa threshold; (i) 5 Pa above and below (40 and 30 Pa)—to determine the influence of subtle sublethal damage accumulation, (ii) 25 Pa above (60 Pa)—to induce non-tolerable sublethal damage with high confidence, and (iii) 25 Pa below (10 Pa)—to represent a physiological shear control that would be highly tolerable; RBC in vivo survive cyclical exposure to this shear stress for a lifespan of up to ~ 120 d.

For the present study, the shear generator mounted on the inverted microscope was used for continuous monitoring of fresh RBC-PVP suspensions exposed to shear stresses of 10, 30, 40, and 60 Pa over a 300-s duration. Videos were captured through a 40 × objective lens (LUCPLFLN 40 × /0.60) at 10 frames per second, and the acquired videos were analysed for average RBC elongation index (EI) in shear, total RBC counts, and ‘abnormal’ RBC counts (i.e., defined as RBC with non-symmetrical morphology—*detail provided below*).

### RBC deformability

The deformability of RBC in shear flow was determined through manual analysis of single cells at specific time intervals across the 300-s shear duration. Given RBC are ellipsoidal in shape when deforming in a viscous medium, RBC deformation was determined by measuring the length of the major (*A*) and minor (*B*) axes to calculate an *EI*, using the equation: *EI* = *(A − B)/(A* + *B).*

### Detection of asymmetrical RBC

**Figure 1:**
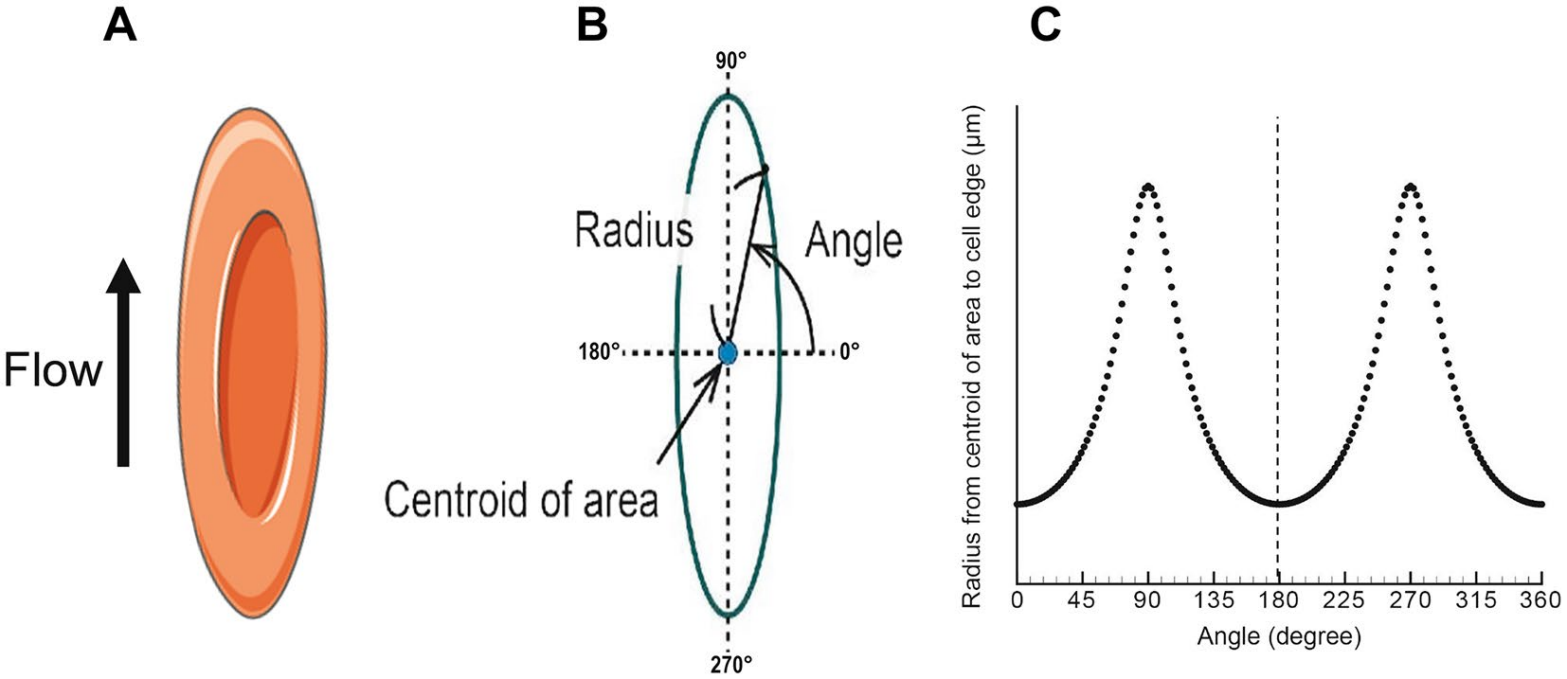
Proposed method for red blood cell (RBC) asymmetrical analysis: Following identification of RBC in shear flow (**A**), each cell must be binarised, edges detected, and analysed for centroid identification (**B**). The radius from the centroid is subsequently determined for each angle around the circumference of the cell and plotted (**C**); the central nadir is mathematically determined by splitting the dataset into left and right sides, each half is analysed for asymmetry by determining difference in area under the curve. For cells with theoretically “perfect” symmetry, this value would be zero.

Before initiation of experimentation, care was taken to optimise the recording environment by setting microscope objective focal distances, enhancing contrast (for improved RBC edge visualisation), and adjusting light intensity for uniform image quality. These pre-processing steps reduced the need for post-processing and thus also the complexity of image analysis. Following image capture, to identify and detect RBC for symmetrical analysis, an image analysis process was developed. Firstly, background illumination of each frame was corrected using the rolling-ball algorithm, and cell edges were made more prominent by enhancing image contrast. With deployment of the Sobel edge detector, large changes in pixel intensity were able to isolate outlines of each cell in frame. To minimise influence of small noise particles prior to cell counting, a Gaussian smoothing filter was applied (*σ* = 5). Cell counting was performed by multi-point selection at each calculated local maxima in the image, where each maxima required at least a prominence of 50 over the noise threshold to be accepted. Each point identified the location of a single cell. The sum of the points (cells) in each frame could then be calculated and exported to a time-matched array. Images of single cells could also be segmented and extracted from the original image. Asymmetry analysis was performed through the following process: 1. each individual cell was rotated to vertically align the cell’s apex (Fig. 1A) before the exported image was binarised through thresholding of each image’s histogram. Cell edges were then segmented from the background, and each cell was analysed for centroid identification (Fig. 1B). An edge detection routine was performed searching outward from the centroid for each angular degree in a counterclockwise direction around the circumference of the cell and plotted (Fig. 1C). The central nadir of the plot was mathematically determined, splitting the dataset into two peaks (*A*_1_ and *A*_2_). Both peaks were analysed for comparative symmetrical deviation by determining the absolute relative difference in the calculated area under each curve (i.e., |{*A*_1_ − *A*_2_}/*A*_1_ × 100|). For cells with greater symmetry, this value approaches zero. A critical threshold of symmetrical RBC was determined by $\bar x + 2\sigma$ of ellipsoidal cells.

### Statistical analysis

All data were analysed with commercially available software (MATLAB; MathWorks; ImageJ, National Institutes of Health; Prism 9, GraphPad Software).

Normality testing was investigated for each dataset using Shapiro–Wilk and Kolmogorov–Smirnov normality tests, with confirmation via inspection of Q–Q plots. RBC deformability (i.e., the elongation index of individual cells) in each shear condition was identified to be non-parametric, thus group comparisons across time were performed using the Kruskal–Wallis test with multiple comparisons. Symmetrical variation for normal and abnormal shaped RBC were identified to have parametric distributions, thus comparisons were performed using an independent samples t-test. Significance was determined at an alpha of 0.05 for all comparative measures. Data is presented as mean ± standard error unless otherwise stated.

## Results

### Micrography of cells in shear

**Figure 2:**
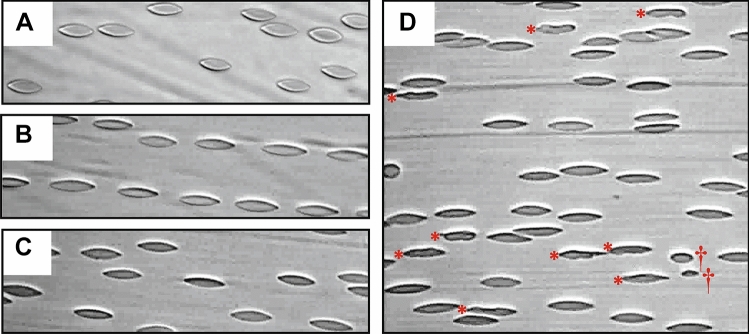
Micrograph of red blood cells (RBC) in shear flow instantaneously imaged at 300 s of exposure to 10 Pa (**A**), 30 Pa (**B**), 40 Pa (**C**), and 60 Pa (**D**). RBC with abnormal morphology (*) and RBC fragments/extracellular vesicles (†) can be observed.

Micrographs of RBC in shear flow are illustrated in Fig. 2. While only normal ellipsoidal RBC are observed at the 300-s timepoint of exposure to 10, 30, and 40 Pa (Fig. 2A–C), the 60 Pa shear condition (Fig. 2D) induced RBC with ‘unstable’ abnormal morphology and increased the presence of cell fragments/extracellular vesicles.

### RBC deformability

**Figure 3:**
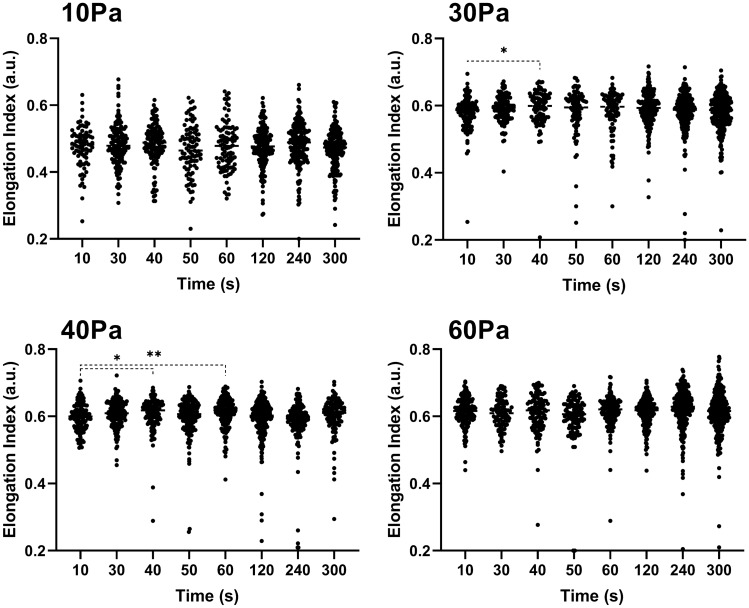
The elongation index of visualised red blood cells exposed to 10, 30, 40, and 60 Pa over the 300-s duration. *p < 0.05. **p < 0.01.

The EI of individually analysed RBC is presented in Fig. 3 for the 300-s duration of each shear condition of the present study. Within each shear condition, the median EI value of all analysed cells at each time point did not significantly decrease.

### RBC asymmetry analysis

**Figure 4:**
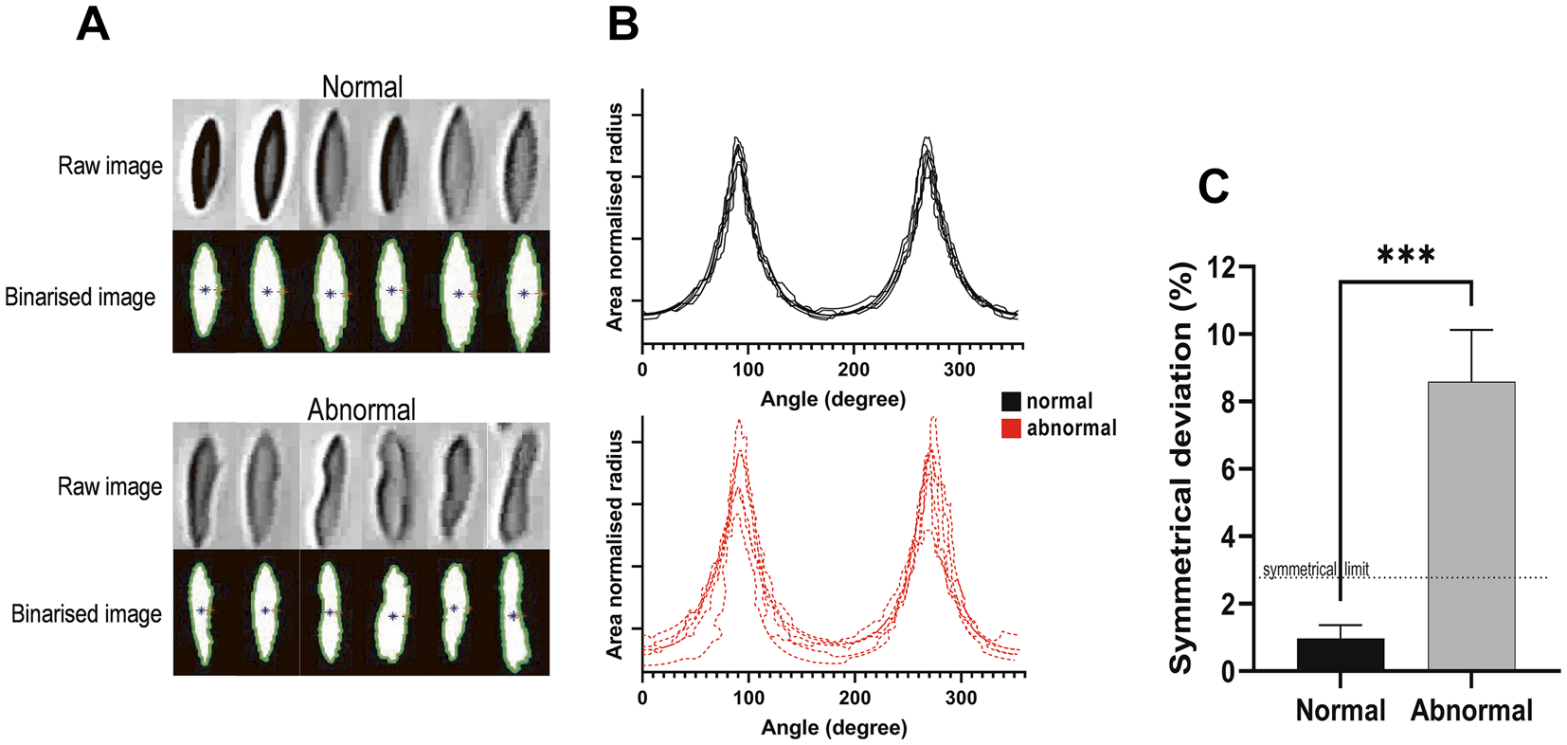
Asymmetry analysis for red blood cells (RBC) identified to have normal and abnormal morphology in shear flow. Following identification of a cell, each cell was binarised, edges detected, and centroid identified (**A**). The radius from the centroid to the cell’s edge was measured around the circumference (**B**). Comparative symmetrical variation significantly differed between normal and abnormal RBC (**C**). The symmetrical upper limit at 2.77% represents $\bar x + 2\sigma$ for normal symmetrical deviation. ***p < 0.001.

Using RBC from the 60 Pa shear condition identified to have representative normal and abnormal morphology, symmetrical analysis was performed on a subset of cells and presented in Fig. 4. Cell identification, segmentation, and binarisation of RBC is illustrated in Fig. 4A for RBC with normal and abnormal morphology. The area-normalised radius from the cell’s centroid to the edge was determined around the circumference and plotted (Fig. 4B). For each individual plot, the central nadir was identified and symmetrical deviation between the comparative area under the curve of left- and right-peaks was calculated (Fig. 4C). The upper limit of symmetrical RBC ($\bar x + 2\sigma$ of normal cells) was determined to be 2.77%; thus, cells with symmetrical variation above this threshold were determined to be asymmetrical. RBC with abnormal morphology had significantly increased symmetrical variation of area under the curve compared with normal ellipsoidal RBC (*p* < 0.001).

### Number of asymmetrical RBC in 60 Pa shear flow

**Figure 5:**
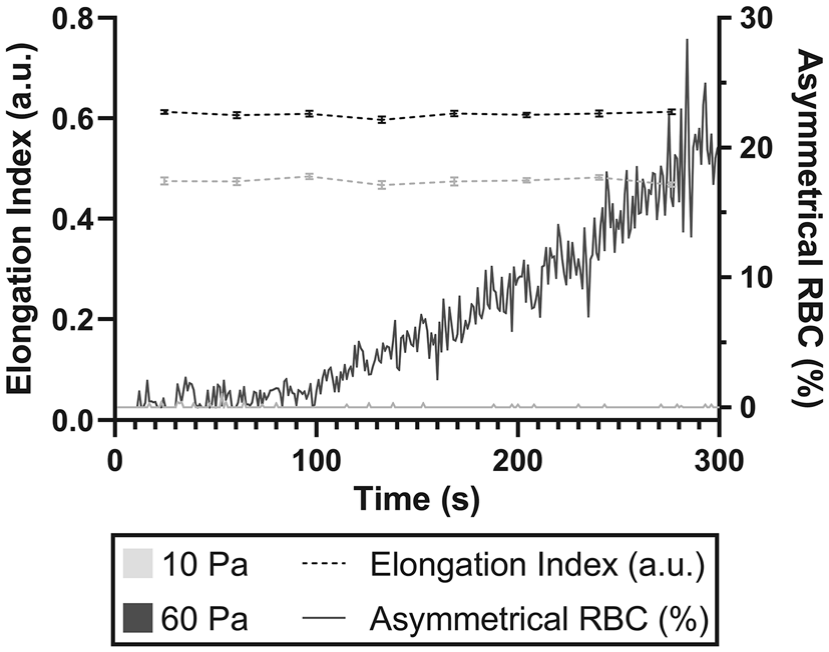
Elongation index and frequency of asymmetrical RBC in 10 and 60 Pa shear conditions. While the ensemble average elongation index remains unchanged for both conditions, the fraction of asymmetrical RBC in the 60 Pa increases beyond ~ 100 s of cumulative exposure, while the 10 Pa condition remains unchanged. Asymmetrical RBC are presented as a percentage of total cell counts per frame.

Following RBC exposure to each shear condition, only the 60 Pa sublethal condition exhibited substantial increases in RBC with altered morphology over the 300-s duration. The ensemble average of RBC elongation index and the percentage of RBC with detected abnormal morphology in the sublethal 60 Pa shear condition and physiological 10 Pa control are presented in Fig. 5. While mean EI remained unaltered across the 300-s duration for both conditions, by contrast to the 10 Pa physiological control with no asymmetrical RBC, the fraction of RBC with asymmetrical morphology in the sublethal 60 Pa condition substantially increased after ~ 100 s of continuous shear exposure.

## Discussion

In the present study, RBC morphological responses to supraphysiological shear stress between 10 and 60 Pa were recorded in a counter-rotating shear generator over 300 s. The salient findings were: (i) the average EI of RBC in shear flow did not change throughout the course of any shear condition; (ii) RBC with abnormal morphology presented only in the 60 Pa condition, with their occurrence accumulating after ~ 100 s of exposure; (iii) the image analysis method we employed for detection of abnormal RBC through (a)symmetrical analyses distinguished between cells with normal and abnormal morphology. These findings collectively indicate that detection of asymmetrical morphology of RBC may provide a sensitive marker to delineate the onset of sublethal blood damage in real-time viscous shear flow. This method of structural analysis may yield insights into the mechanistic process of mechanically induced RBC deterioration/fragmentation.

It is well known that the biophysical properties of RBC are sensitive to chemical assault^37^, physiological aging^31^, and accumulated stress–strain history^38,39^, yet in the present study, traditional methods of assessing EI (deformability) in shear flow remained unchanged, even following the onset of detectable cell fragments. Prior reports of RBC EI in lower shear flows ≤ 20 Pa (assessed with indirect ektacytometry) are congruent with the unchanged EI response presented in Fig. 3—especially the 10, 30, 40 Pa condition^40^. In the 60 Pa shear condition however, even following marked increases in asymmetrical and fragmented cells, still no change in group EI responses were detected. Given ektacytometry generates a single output from laser diffractometry, this technique has been identified to be vulnerable to the presence of mixed subpopulations of biophysically altered cells^41^, highlighting the methodological benefits of direct visual inspection for RBC subpopulation assessment. While ektacytometry was not used in the current study, the presence of morphologically altered cell populations able to be observed in Fig. 2 (with narrowed central region and artificially smaller ellipse short axes) could potentially induce increased group EI responses from laser diffractometry and partly explain the transiently increased EI-stability previously reported in 64 and 100 Pa shear flows^7,12^. Moreover, given greater shear history accumulation likely induces the onset of substantial fragmentation of these morphologically aberrant cells, subsequent RBC counts are predicted to eventually decrease, which has been reported to decrease group EI values^42^. Collectively the observed changes in RBC morphology may partly explain the biphasic grouped EI response previously reported in studies employing ektacytometry.

In contrast with traditional RBC ektacytometry investigations adopting stress–strain curve assessment following preconditioning shear treatment, considerably longer exposure duration was required for the onset of shape-altered RBC (Fig. 5). At comparable shear exposure to the present study, Simmonds and Meiselman^7^ were able to first detect significant cell rigidification following 64 Pa for only 4 s, with longer duration conditioning times resulting in exacerbated rigidification. The stress–strain assessment of these RBC identified that impaired RBC mechanics were most evident during subsequent exposure to lower shears (typically within physiological ranges of ≤ 10 Pa), while RBC mechanics tended to revert towards expected values when examined under 16+ Pa. Our direct visualisation approach tends to support this prior report that employed ektacytometry, and we hypothesise that greater accumulation of non-tolerable shear (i.e., increased shear magnitudes or durations of exposure) would likely increase rates and counts of asymmetric RBC, prior to the onset of haemolysis. While not the focus of the present investigation, it is plausible that initial shear-induced (over)stretching of RBC may propagate phospholipid translocation^19,22^, destruction of spectrin-membrane junctional attachment sites^43^, and forced unfolding of spectrin networks causing internal skeletal fragmentation^44,45^. Collectively, these biophysical alterations to RBC may partly separate the external lipid bilayer (still able to deform) from internal structural architecture of the cell, inducing the observed asymmetric morphology and altering subsequent low-shear stability as evidenced through increased cell tumbling, and decreased alignment and elongation; at least within successive physiological ranges of shear^39^. To observe this speculated cell mechanics, following exposure to the 60 Pa shear condition, a qualitative extension was performed where 1 Pa of shear stress was subsequently applied; substantial cell tumbling, variation in size/morphology, cell blebbing, and RBC aggregates could easily be observed (see supplementary video 1). In the present study, if the continuous shear bouts had instead been intermittent, it is likely that the low shear rigidification observed in other studies would have been detected well before the onset of morphological alterations. Nevertheless, both the low shear rigidification and morphological alteration are likely part of the same haemolytic process of shear-induced division/fragmentation. Deeper mechanistic inspection of mechanobiological/shear induced structural weaknesses of RBC would be of value, given potential targets for interventional protection or damage modulation may be identified.

Although the shear stress conditions utilised in the present study were limited to supraphysiological shears that are reported not to induce significant increases in plasma free haemoglobin (i.e., haemolysis), an increase in RBC-derived microparticles was identified following prolonged exposure to 60 Pa. Such microparticle increase in a ‘subhaemolytic environment’ has been recently reported^17^; given cell fragmentation represents lethal deterioration of RBC, perhaps the haemolytic threshold determined by conventional assessment of RBC destruction measured via free haemoglobin requires reconsideration. Indeed, release of cytosolic haemoglobin into surrounding media represents end-point cell destruction; however, our data suggest that bulk fragmentation (a lethal transition for RBC) can still be observed in the ‘subhaemolytic’ 60 Pa condition. Thus, given the presented complexity of the RBC deterioration process, reconsideration of defining characteristics of marked cell destruction, and which domains should be categorised as ‘lethal/sublethal’ require review. By contrast to previously reported haemolytic thresholds^3–5,46^, the present study advocates for consideration and implementation of more sensitive indicators of RBC damage.

To detect damaged RBC in real-time shear flow, the current study proposed a new image analysis method to delineate abnormal RBC though ellipsoidal symmetrical assessment. As illustrated in Fig. 4, the angle series of RBC shape data using area-normalised radius of individual cells was successfully able to separate abnormal cells that had transgressed from control morphology. Inclusion of target area standardisation of radial measurements enhances the versatility of such an approach for future potential industrial use, by enabling uptake on various systems with different levels of microscopic magnification, elongation due to shear, and cell types/volume. The proposed method of cell analysis may also enable more accurate automated techniques for shape/morphology assessment (at stasis and in flow) able to quickly evaluate levels of haemopathology (e.g. level of echinocytosis or severity of sickle cell). It should be noted that while the 30 mPa·s viscosity solution in the current study facilitated the specific requirements for accurate shear quantification and control, the approach for shape assessment in flow may be limited in lower (more physiologically relevant) viscosity solutions where the viscosity ratio of RBC cytosol to plasma will be > 1.

## Conclusion

The current study successfully visualised the onset of RBC morphological deterioration through asymmetric elongation under sublethal supraphysiological shear stress. While the majority of RBC remained ellipsoidal and symmetrical under all shear conditions, following prolonged exposure to 60 Pa, ~ 20% of asymmetrical fragmentating RBC were detected. Our newly proposed image analysis method of symmetrical assessment was able to detect the presence of abnormal cells and may provide the foundations for future systems for rapid automated detection of sublethal blood trauma.

Although the complex shear profiles within cardiovascular devices differ drastically from our simplified shear flow conditions in the current study, it is likely that similar morphological abnormalities and fragments will still present in the small subregions that exert extreme magnitudes of shear stress onto subpopulations of blood.

## Supplementary Information

Supplementary Video 1.
